# A WALK-THROUGH DEMENTIA: UNDERSTANDING ALZHEIMER’S DISEASE OR RELATED DEMENTIA THROUGH YOUTUBE VR VIDEOS

**DOI:** 10.1093/geroni/igae098.2578

**Published:** 2024-12-31

**Authors:** Xiaoli Li, Xiaoyu Liu, Eman Alanazi, Arianna Goss

**Affiliations:** Southern Illinois University Carbondale, Cabondale, Illinois, United States; Saint Louis University, St. Louis, Missouri, United States; Saudi Electronic University, Riyadh, Ar Riyad, Saudi Arabia; Saint Louis University, St. Louis, Missouri, United States

## Abstract

Emerging research has highlighted the potential of Virtual Reality (VR) as a tool for raising awareness of Alzheimer’s disease or related dementia (ADRD) among healthcare students and professionals. However, there is limited VR research focused on engaging the general public. This research aimed to examine the issues faced by YouTube users who interacted with the VR application “A Walk-Through Dementia” by analyzing their posts. We collected 12,754 comments from VR videos ‘A Walk-Through Dementia’, which simulate the everyday challenges faced by individuals with ADRD, providing viewers with an immersive experience on the condition. We conducted topic modeling to gauge viewer opinions and reactions to the videos. A pre-trained BERT model was utilized to transform YouTube comments into high-dimensional vector embeddings, enabling systematic identification and detailed analysis of principal topics and their intrinsic thematic structures within the dataset. Our analysis identified five distinct topics. The predominant topic, reflected in 2,917 comments, focused on users’ personal experiences regarding the impact of ADRD on patients and caregivers. The other topics were organized into four main categories: support and empathy for individuals affected by ADRD, the absence of caregiver, unfriendly environment, and positive reactions to the VR experience. With the technique of topic modeling, the present study indicated that the VR application is an engaging and experiential learning tool that can provide public with deeper awareness of living with ADRD. Future research need explore more VR applications on social media as it has the potential to reach broader audiences that disseminate knowledge of ADRD.

